# Outcome Prediction in Mathematical Models of Immune Response to Infection

**DOI:** 10.1371/journal.pone.0135861

**Published:** 2015-08-19

**Authors:** Manuel Mai, Kun Wang, Greg Huber, Michael Kirby, Mark D. Shattuck, Corey S. O’Hern

**Affiliations:** 1 Department of Physics, Yale University, New Haven, Connecticut, United States of America; 2 Department of Mathematics, Colorado State University, Fort Collins, Colorado, United States of America; 3 Department of Mechanical Engineering and Material Science, Yale University, New Haven, Connecticut, United States of America; 4 Kavli Institute for Theoretical Physics, Kohn Hall, University of California Santa Barbara, Santa Barbara, California, United States of America; 5 Department of Computer Science, Colorado State University, Fort Collins, Colorado, United States of America; 6 Benjamin Levich Institute and Physics Department, The City College of New York, New York, New York, United States of America; 7 Department of Applied Physics, Yale University, New Haven, Connecticut, United States of America; 8 Graduate Program in Computational Biology and Bioinformatics, Yale University, New Haven, Connecticut, United States of America; University of Glasgow, UNITED KINGDOM

## Abstract

Clinicians need to predict patient outcomes with high accuracy as early as possible after disease inception. In this manuscript, we show that patient-to-patient variability sets a fundamental limit on outcome prediction accuracy for a general class of mathematical models for the immune response to infection. However, accuracy can be increased at the expense of delayed prognosis. We investigate several systems of ordinary differential equations (ODEs) that model the host immune response to a pathogen load. Advantages of systems of ODEs for investigating the immune response to infection include the ability to collect data on large numbers of ‘virtual patients’, each with a given set of model parameters, and obtain many time points during the course of the infection. We implement patient-to-patient variability *v* in the ODE models by randomly selecting the model parameters from distributions with coefficients of variation *v* that are centered on physiological values. We use logistic regression with one-versus-all classification to predict the discrete steady-state outcomes of the system. We find that the prediction algorithm achieves near 100% accuracy for *v* = 0, and the accuracy decreases with increasing *v* for all ODE models studied. The fact that multiple steady-state outcomes can be obtained for a given initial condition, *i.e.* the basins of attraction overlap in the space of initial conditions, limits the prediction accuracy for *v* > 0. Increasing the elapsed time of the variables used to train and test the classifier, increases the prediction accuracy, while adding explicit external noise to the ODE models decreases the prediction accuracy. Our results quantify the competition between early prognosis and high prediction accuracy that is frequently encountered by clinicians.

## Introduction

The immune response to infection is a complex process that involves a wide range of length scales from proteins to cells [1–4], tissues [5], and organ systems [6]. Despite enormous progress over the past 30 years in developing mathematical models for the immune response to infectious disease such as tuberculosis [7, 8], HIV [9–13], and influenza [14, 15], these models still have not been able to dramatically improve patient diagnosis, prognosis, and treatment [16, 17]. Instead, vaccine and drug development often relies on costly trial-and-error methods [18]. However, advances in gene sequencing capabilities [19], increasing speeds of computer processors, and the ability to store enormous amounts of medical data promise dramatic improvements in mathematical approaches to predicting and controlling the response to infectious disease [20–23].

One promising mathematical approach is to use machine learning methods on large data sets to classify patients as healthy or sick, perform early warning analyses for early detection of infection, or identify the minimal set of genes responsible for a particular immune response.[24, 25] However, many questions are left unanswered in such studies. For example, how much and what kinds of data are required to have confidence in the machine learning predictions and what are the underlying biophysical mechanisms for the relationships between variables that are identified by these techniques? Further, it is difficult to determine differences in the immune response that arise from patient-to-patient variations compared to slight differences in the initial conditions of each patient.

In this manuscript, we focus on sets of ordinary differential equations (ODEs) as mathematical models for the immune response to infection. The advantages of ODEs are manifold: 1) Each ‘virtual patient’ can be considered as a set of parameters in the set of ODEs; 2) There is essentially no limit on the amount of data that can be collected on each virtual patient; 3) The accuracy of machine learning predictions can be explicitly tested as a function of the number of time points and initial conditions for each patient and the number of patients included in the training and testing sets; and 4) analysis of the fixed points (or steady-state outcomes) and basins of attraction of the ODEs can give biophysical insight into the immune response to infection.

We will investigate several classes of ODE models for the immune response to infection. First, we will describe a four-dimensional model for the acute inflammatory response to a pathogen load that was studied in detail in Ref. [26]. We will then consider reduced versions of this model with fewer variables and parameters obtained by slaving one or more of the original four variables, as well as changes to the form of the ODEs that alter the fixed point structure and flows between them. For each model, a virtual patient is defined by one set of parameters. Given an initial condition (values of the variables at time *t* = 0), the patient will evolve deterministically to one of several possible discrete steady-state (*t* → ∞) outcomes, or fixed points. Thus, for each patient, we can determine the basins of attraction that map initial conditions for all of the variables to steady-state outcomes by numerically integrating the sets of ODEs.

**Fig 1:**
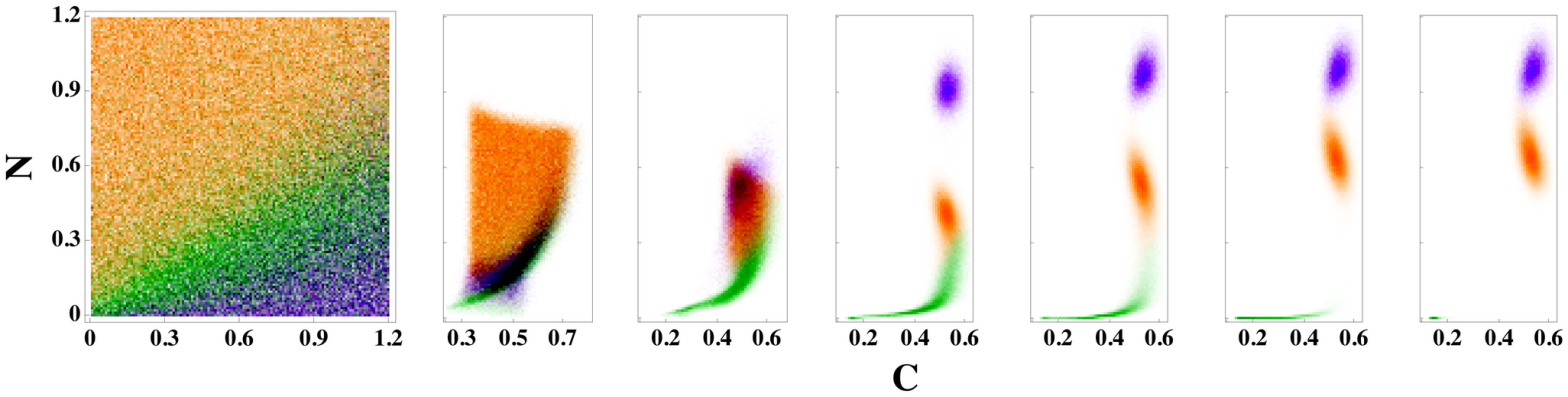
Time evolution of patient outcomes for a range of neutrophil (*N*) and cortisol (*C*) initial conditions for model (1). (left) Patient outcomes in the long-time limit given *N* and *C* initial conditions for model (1) are shaded green, orange, and purple for the steady-state outcomes of health, aseptic, and septic death, respectively. The initial values of the pathogen load and damage are *P*_0_ = 0.35 and *D*_0_ = 0, and patient variability is set to *v* = 5%. The right six panels indicate how the systems in the leftmost panel separate in the *N* and *C* plane as time increases, *t* = 10, 20, 50, 100, 250, and 500, from left to right.

We seek to determine the limits of the prediction accuracy of discrete steady-state outcomes of ODEs as a function of patient variability (*i.e.* random fluctuations in parameter values) using machine learning techniques. In the limit of zero patient variability, our simple classification algorithm (logistic regression) can achieve nearly perfect prediction accuracy even when the classification occurs on variables at short times. However, as the patient variability increases, the basins of attraction for different patients yield different outcomes for a given initial condition as shown in Fig 1 for 5% patient variability in model (1) for the immune response to infection. (See Materials and Methods). The fact that each set of initial conditions does not possess a unique outcome places a fundamental limit on the predictability of patient outcomes. Thus, we find that the machine learning prediction accuracy decreases with increasing patient variability. In contrast, for a given patient variability, the prediction accuracy increases with the time used for classification as the systems converge to their steady-state outcomes (Fig 1). We also show that at short times our classification algorithm saturates the theoretical limit for the prediction accuracy in the presence of patient variation, and that the addition of external noise only worsens the outcome prediction accuracy.

The manuscript is organized as follows. In the Materials and Methods section, we introduce several ODE systems that have been used to model the host immune response to infection [26], including their parameter sets and discrete steady-state outcomes, and describe how we implement patient variability in the ODE models. We also describe the logistic regression classification algorithm that we implement to predict steady-state outcomes and measures of the performance of the classification algorithm. In the Results section, we emphasize our three main results that hold for all of the ODE models we studied: 1) patient variability leads to overlap of the basins of attraction for the steady-state outcomes, which limits the outcome prediction accuracy, 2) the prediction accuracy increases with the time used for classification because the basins of attraction separate with increasing time, and 3) the addition of external measurement noise further reduces the prediction accuracy. In the Discussion section, we point out the clinical implications of our work and describe important future studies of the prediction accuracy for ODE models with continuous, rather than discrete, steady-state outcomes.

## Materials and Methods

Our studies focus on several ODE models with varying complexity for the immune response to pathogen load that were first introduced in Ref. [26]. These ODE models can have up to four coupled variables that represent the concentration of pathogen *P*, activated neutrophils *N*, inflammation (or damage) *D*, and immuno-suppressor (cortisol) *C*. The models include interactions between these four quanties. For example, the presence of pathogen *P* > 0 causes an immune response, where neutrophils are activated and *N* increases. Neutrophils kill pathogen, which decreases *P*, but also cause inflammation (damage), which increases *D*. The cortisol level *C* increases when there is a high neutrophil level, which then reduces the neutrophil level.

Model (1) (Eqs 1–4) includes all four variables *P*, *N*, *D*, and *C*. The right-hand side of *dP*/*dt* is a sum of three terms. The first term enables logistic growth of the pathogen. In the absence of any other terms, any positive initial *P*_0_ will cause *P* to grow logistically to the steady-state value *P*_∞_. The second term mimics a local, non-specific response to an infection. For small values of *P*, the decrease is proportional to *P*. For larger values of *P*, the decrease caused by the second term is constant. The third term models the decrease of *P* due to interactions with activated immune cells (neutrophils) *N*. Activated neutrophils *N* can directly decrease *P*. The anti-inflammatory response, which is captured by the cortisol level *C*, mitigates this effect leading to a decrease of *P* proportional to N*P/(1 + (*C*/*C*_∞_)^2^).

Two terms determine the rate of change in neutrophils, *dN*/*dt*. The first term accounts for the fact that neutrophils can be activated if a resting neutrophil cell encounters a pathogen *P* or an already activated neutrophil *N*. Furthermore, tissue damage *D* also triggers the activation of neutrophils. The second term describes the death of neutrophils *N*, with the decrease in *N* proportional to the amount of neutrophils present.

The rate of change in damage *dD*/*dt* is also controlled by two terms. The first term mimics positive feedback between *D* and *N*. Activated phagocytes cause collateral damage in the tissue. Again, the effectiveness of *N* is mitigated by the anti-inflammatory response 1/(1 + (*C*/*C*_∞_)^2^). The saturation function *f*_*s*_ models the fact that the effect of *N* on *D* saturates for large *N*. The second term, -*μ*_*d*_ *D*, represents repair of the tissue.

The anti-inflammatory response *C* increases with the source term *s*_*c*_. In addition, there is a natural death rate *μ*_*c*_, which leads to a positive steady-state value of *C* in the absence of any immune activation *N* or damage *D*. However, even small amounts of damage and neutrophils will up-regulate *C*. In the case of small *N* + *k*_*cnd*_ *D*, the production of *C* is proportional to *N* + *k*_*cnd*_ *D*, while for large values of *N* + *k*_*cnd*_ *D*, changes in *C* are proportional to *k*_*cn*_. Again, the effectiveness of *N* is mitigated by 1/(1 + (*C*/*C*_∞_)^2^).

Model (1) has 21 parameters: *k*_*pm*_, *k*_*mp*_, *s*_*m*_, *μ*_*m*_, *k*_*pg*_, *P*_∞_, *k*_*pn*_, *k*_*np*_, *k*_*nn*_, *s*_*nr*_, *μ*_*nr*_, *μ*_*n*_, *k*_*nd*_, *k*_*dn*_, *x*_*dn*_, *μ*_*d*_, *C*_∞_, *s*_*c*_, *k*_*cn*_, *k*_*cnd*_, and *μ*_*c*_ with units provided in Table 1. Depending on the values of these parameters, model (1) possesses different numbers of fixed points with varying stabilities. However, we will focus on a specific parameter regime (given in Table 1) with three stable fixed points, which correspond to the physiological steady-state outcomes: health, septic death, and aseptic death.

**Table 1:** Mean values and units for the ODE parameters. The mean values *μ*_*q*_ and their units for the the twenty-three parameters *q* for models (1)-(6). [*y*] indicates the dimension of *y* and time is measured in hours (*h*).

Parameter *q*	Mean Value *μ*_*q*_	Units
*k*_*pm*_	0.6	[*M*]^−1^ *h* ^−1^
*k*_*mp*_	0.01	[*P*]^−1^ *h* ^−1^
*s*_*m*_	0.005	[*M*] *h* ^−1^
*μ*_*m*_	0.002	*h* ^−1^
*k*_*pg*_	0.6	*h* ^−1^
*P*_∞_	20.0	[*P*]
*k*_*pn*_	1.8	*h* ^−1^
*k*_*np*_	0.1	[*N*] [*P*]^−1^
*k*_*nn*_	0.0	1 [*N*]^−1^ *h* ^−1^
*s*_*nr*_	0.08	[*N*] *h* ^−1^
*μ*_*nr*_	0.12	*h* ^−1^
*μ*_*n*_	0.05	*h* ^−1^
*k*_*nd*_	0.02	[*D*]^−1^ *h* ^−1^
*k*_*dn*_	0.35	[*D*] *h* ^−1^
*x*_*dn*_	0.06	[*N*]
*μ*_*d*_	0.02	*h* ^−1^
*C*_∞_	0.28	[*C*]
*s*_*c*_	0.0125	[*C*] *h* ^−1^
*k*_*cn*_	0.04	[*C*] *h* ^−1^
*k*_*cnd*_	48.0	[*N*] [*D*]^−1^
*μ*_*c*_	0.1	*h* ^−1^
*k*	1	[*x*]^−1^
*B*	1	[*x*]

Model (1)

$$\frac{dP}{dt}=\;k_{pg}P(1-\frac{P}{P_{\infty }})-\frac{k_{pm}s_{m}P}{\mu_{m}+k_{mp}P}-k_{pn}f\left(N,C\right)P$$ (1)

$$\frac{dN}{dt}=\;\frac{s_{nr}R}{\mu_{nr}+R}-\mu_{n}N$$ (2)

$$\frac{dD}{dt}=\;k_{dn}f_{s}(f(N,C))-\mu_{d}D$$ (3)

$$\frac{dC}{dt}=\;s_{c}+\frac{k_{cn}\;f\left(N+k_{cnd}D,C\right)}{1+f(N+k_{cnd}D,C)}-\mu_{c}C,$$ (4)

where

$$R=f(k_{nn}N+k_{np}P+k_{nd}D,C),$$ (5)

$$f\left(V,C\right)=V/\left(1+{\left(C/C_{\infty }\right)}^{2}\right),$$ (6)

$$f_{s}\left(V\right)=V^{6}/\left(x_{nd}^{6}+V^{6}\right).$$ (7)

Models (2)-(5) given below are simplified versions of model (1). A summary of the dimension, number of parameters, and number of stable fixed points for each of these ODE models is shown in Table 2. To obtain model (2) from (1), *C* is set to a constant $\bar{C}=0.23$ and the remaining terms define a three-variable model with *P*, *N*, and *D*. For model (3), we set $C=\bar{C}$ and *D* = 0, which gives a two-variable model for *P* and *N*. For model (4), we set $C=\bar{C}$ and *P* = 0 to obtain a two-variable model for *N* and *D*. In this model, the value of the initial rise in *N* can be thought of as the response to trauma. For model (5), we set $C=\bar{C}=0.1$, *D* = 0, and *N* = 0, which gives a one-dimensional model for *P*. This model only treats the innate immune response with no activated neutrophils.

**Table 2:** Summary of the ODE models. The dimension, number of parameters, number of stable fixed points, and values for the key parameters *k*_*pg*_ and $\bar{C}$ for models (1)-(6).

Model	Dimension	Parameters	Stable Fixed Points	*k*_*pg*_	$\bar{C}$
1	4	21	3	0.6	N/A
2	3	18	3	1.2	0.23
3	2	14	2	1.2	0.23
4	2	8	2	1.2	0.23
5	1	6	2	0.6	0.1
6	1	2	2	N/A	N/A

Model (2)

$$\frac{dP}{dt}=\;k_{pg}P(1-\frac{P}{P_{\infty }})-\frac{k_{pm}s_{m}P}{\mu_{m}+k_{mp}P}-k_{pn}f\left(N,\bar{C}\right)P$$ (8)

$$\frac{dN}{dt}=\;\frac{s_{nr}R}{\mu_{nr}+R}-\mu_{n}N$$ (9)

$$\frac{dD}{dt}=\;k_{dn}f_{s}(f(N,\bar{C}))-\mu_{d}D$$ (10)

Model (3)

$$\frac{dP}{dt}=\;k_{pg}P(1-\frac{P}{P_{\infty }})-\frac{k_{pm}s_{m}P}{\mu_{m}+k_{mp}P}-k_{pn}f\left(N,\bar{C}\right)P$$ (11)

$$\frac{dN}{dt}=\;\frac{s_{nr}R_{3}}{\mu_{nr}+R_{3}}-\mu_{n}N,$$ (12)

where

$$R_{3}=f\left(k_{nn}N+k_{np}P,\bar{C}\right).$$ (13)

Model (4)

$$\frac{dN}{dt}=\;\frac{s_{nr}R_{4}}{\mu_{nr}+R_{4}}-\mu_{n}N$$ (14)

$$\frac{dD}{dt}=\;k_{dn}f_{s}(f(N,\bar{C}))-\mu_{d}D,$$ (15)

where

$$R_{4}=f\left(k_{nn}N+k_{nd}D,\bar{C}\right).$$ (16)

Model (5)

$$\frac{dP}{dt}=k_{pg}P(1-\frac{P}{P_{\infty }})-\frac{k_{pm}s_{m}P}{\mu_{m}+k_{mp}P}$$ (17)

**Fig 2:**
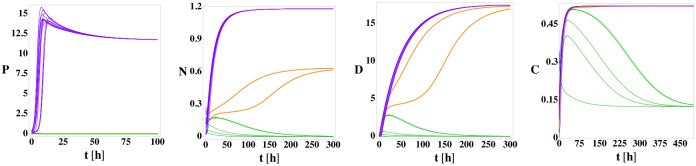
*P*, *N*, *D*, and *C* versus time for model (1) from 20 random initial conditions with no patient variation. For the parameter values in Table 1 but with *k*_*pg*_ = 1.2, model (1) possesses three fixed points: health (green lines), septic death (purple lines), and aseptic death (orange lines). The initial conditions are sampled randomly within the cube: 0 ≤ *P*_0_ ≤ 0.42, 0 ≤ *N*_0_ ≤ 0.255, *D*_0_ = 0, and 0 ≤ *C*_0_ ≤ 0.35. The three fixed points can be differentiated by the steady-state values of *P* and *D*: health (*P* = 0, *D* = 0), aseptic death (*P* = 0, *D* > 0), and septic death (*P* > 0, *D* > 0). 5, 13, and 2 of the initial conditions evolve to the health, septic death, and aseptic death fixed points, respectively.

In Fig 2, we show the time evolution of the four variables *P*, *N*, *D*, and *C* for model (1) for twenty different sets of random initial conditions to illustrate its three stable fixed points (health, septic death, and aseptic death) using the parameter values in Table 1. For trajectories that approach the septic death fixed point, the pathogen and neutrophil levels grow rapidly. The high neutrophil level causes cortisol to increase as well. Despite the high level, the neutrophils cannot reduce the pathogen load and the cortisol level is not large enough to reduce the neutrophil level. As a result, the high neutrophil level causes significant damage at long times, which is termed septic death due to the associated high pathogen level. Thus, the septic death steady-state outcome is characterized by *P* > 0, *N* > 0, *D* > 0, and *C* > *C*_∞_.

In the healthy state, the pathogen level can be reduced to zero by the neutrophils, and the neutrophil level can be reduced to zero by cortisol. Once the neutrophil level is zero, the cortisol level returns to its background level and damage decreases to zero. Thus, the healthy state is characterized by *P* = 0, *N* = 0, *D* = 0, and *C* = *C*_∞_.

During the approach to the aseptic death fixed point, the neutrophil level is strong enough to reduce the pathogen level to zero, but the cortisol level is insufficient to reduce the neutrophil level to zero, which leads to increasing damage. Thus, the aseptic death fixed point is characterized by *P* = 0, *N* > 0, *D* > 0, and *C* > *C*_∞_.

We also studied a generalization of model (5). Model (6) is a one-dimensional ODE for the variable *x* with the same fixed point structure as model (5) (Eq 17), but different locations for the fixed points, which can be obtained by tuning the two parameters *k* and *B*.

Model (6)

$$dx/dt=\{\begin{array}{cc} B\;\cos(k\;x+\pi /2), & \text{if}\;0\leq x\leq 2\pi /k\\ -B\;k\;\sin\left(5\pi /2\right)(x-2\pi /k), & \text{if}\;2\pi /k<x. \end{array}$$ (18)

**Fig 3:**
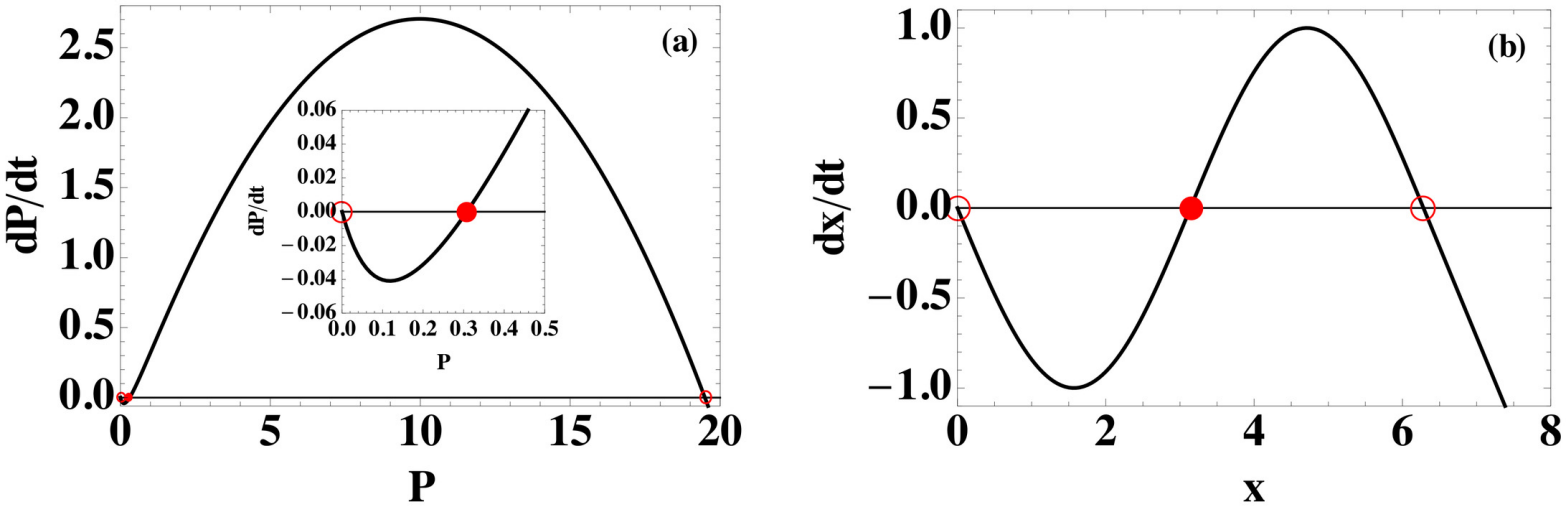
Comparison of ODE models (5) and (6). The functions *f*(*P*) = *dP*/*dt* and *f*(*x*) = *dx*/*dt* for models (a) (5) and (b) (6). (See Eqs (17) and (18)). Stable and unstable fixed points are marked by open and filled circles, respectively. Both models share the same fixed point topology: one stable fixed point at zero and one at a positive value. The unstable fixed point lies between the two stable fixed points. The inset in (a) magnifies *dP*/*dt* near the origin.

Model (5), which is a one-dimensional ODE for pathogen *P*, possesses three fixed points: *P* = 0, 0.3078, and 19.49 for the mean parameters in Table 1. As shown in Fig 3(a), the two outer fixed points for model (5) are stable, and the middle fixed point, which is near zero, is unstable. For model (6), the central unstable fixed point is moved to the midpoint of the two outer stable fixed points and the shape of the function is changed to retain the fixed point structure.

**Fig 4:**
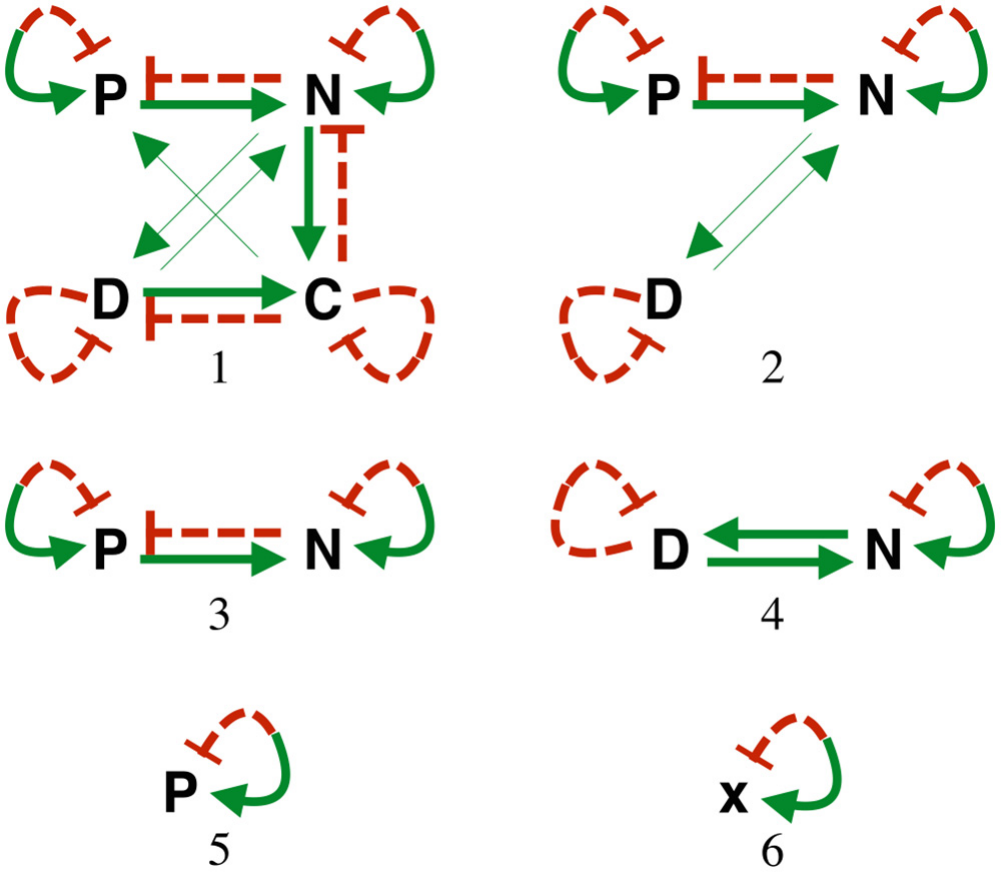
Interactions between variables for models (1)—(6). The green, solid arrows betwen pairs of variables denote upregulation and the red, dashed bars indicate downregulation for ODE models (1)-(6). Combined dashed and solid arrows indicate that either up or downregulation is possible depending on the values of the variables and parameters.

Fig 4 summarizes the interactions between all of the variables in models (1)—(6). Green, solid arrows connecting pairs of variables indicate upregulation, while red, dashed bars indicate downregulation. For all models, both up and down self-regulation can also occur. If the sign of the feedback (*i.e.* positive or negative) depends on the values of the variables and parameters, self-regulation is represented using both an arrow and bar beginning and ending on a single variable.

**Fig 5:**
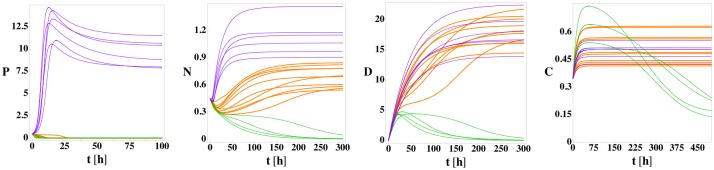
*P*, *N*, *D*, and *C* versus time for model (1) with 20 sets of randomly selected parameters with the same initial conditions. 10% patient variation allows the system to reach the health, aseptic, and septic death fixed points with the initial condition *P*_0_ = 0.45, *N*_0_ = 0.45, *D*_0_ = 0, and *C*_0_ = 0.35, whereas only the septic death fixed point is obtained for this initial condition with no patient variation. 4, 6, and 10 of the trajectories evolve to the health, septic death, and aseptic death fixed points, respectively. The means of the parameters were set to those provided in Table 1, except the mean of *k*_*pg*_ = 0.8 was chosen to display maximum variability of the steady-state outcomes.

The outcomes of the immune response to infection can vary from patient to patient, even with the same initial conditions (*e.g.* the pathogen load) (See Fig 5). To introduce patient variability into the ODE models, we select the parameters ({*q*}) in models (1)-(6) randomly from independent Gaussian distributions with mean values *μ*_*q*_ in Table 1 and standard deviation relative to the mean (or coefficient of variation) *v*. Negative values of the parameters can cause the ODE models to become non-integrable, and thus the parameter distributions are cut off so that the parameter values are non-negative. (We also studied log-normal distributions for the parameters, which ensure that *q* > 0, and found qualitatively similar results). We solve the ODE models (1)-(6) for 10^4^ sets of parameters for each of the 10^4^ random initial conditions at each *v*. The limits for the sampling of the initial conditions for each model are given in Table 3. We then perform classification analyses on these trajectories to predict the steady-state outcomes.

**Table 3:** Summary of the initial condition ranges used in the ODE models. The ranges of the initial conditions for each of the variables in ODE models (1)-(6).

Model	Initial Condition Ranges
1	0 ≤ *P*_0_ ≤ 0.9, 0 ≤ *N*_0_ ≤ 0.33, *D*_0_ = 0, 0 ≤ *C*_0_ ≤ 0.5
2	0 ≤ *P*_0_ ≤ 0.1, 0 ≤ *N*_0_ ≤ 0.15, 0 ≤ *D*_0_ ≤ 0.1
3	0 ≤ *P*_0_ ≤ 0.2, 0 ≤ *N*_0_ ≤ 0.3
4	0 ≤ *N*_0_ ≤ 0.15, 0 ≤ *D*_0_ ≤ 0.1
5	0 ≤ *P*_0_ ≤ 0.7
6	0 ≤ *x*_0_ ≤ 2*π*

The prediction accuracy *A* is defined as the number of correct classifications of the steady-state outcomes divided by the total number of classifications. The accuracy quantifies the quality of the prediction of all of the steady-state outcomes and is therefore a discriminating measure of the overall performance of the classifier. We also evaluated the performance of the classifier separately for each of the predicted steady-state outcomes. For model (1), there are three steady-state outcomes, and thus there are three possible true-positive rates (TPR), one for each outcome. In addition, there are two false-negative rates (FNR) for each outcome. Complete quantification of the performance can be obtained using the 3 × 3 confusion matrix of the classifier. There are several combinations of entries of the confusion matrix that can be plotted against each other. In the Results section, we plot the TPR versus the FNR values as well as the accuracy to assess the performance of the classification analyses.

We employ logistic regression to obtain predictions for the steady-state outcomes. A logistic regression algorithm, in its most basic form, is a method to classify data into two groups. Based on input data $x^{\left(i\right)}=(x_{1}^{\left(i\right)},x_{2}^{\left(i\right)},\ldots ,x_{N}^{\left(i\right)})$, we attempt to predict the class label *y* ^(*i*)^ ∈ {0, 1} (*i.e.* steady-state outcome). In our case, the input data are the model variables at a specified time *t*_*c*_, where *N* is the number of variables in the model. To train the classifier, we employ labeled training data (*x* ^(*i*)^, *y* ^(*i*)^). The logistic regression algorithm then fits a logistic function

$$P_{\theta }\left(x^{\left(i\right)}\right)=\frac{1}{1+e^{\theta_{0}+\theta_{1}\;x_{1}^{i}+\theta_{2}\;x_{2}^{i}+\cdots +\theta_{N}\;x_{N}^{i}}}$$ (19)

to the training data. The parameters *θ*_*j*_, where *j* = 0, 1, 2, …, *N*, are determined by minimizing the cost function

$$J\left(\theta_{0},\theta_{1},\ldots ,\theta_{N}\right)=-\frac{1}{m}[\sum_{i=1}^{m}y^{\left(i\right)}\text{log}\left(P_{\theta }\left(x^{\left(i\right)}\right)\right)+\left(1-y^{\left(i\right)}\right)\text{log}\left(1-P_{\theta }\left(x^{\left(i\right)}\right)\right)],$$ (20)

where *m* is the number of training samples. Evaluating *P*_*θ*_(*x* ^(*k*)^) on an unseen data point *x* ^(*k*)^ gives the probability that *y* ^(*k*)^ = 1. If *P*_*θ*_(*x* ^(*k*)^) is greater than some threshold 0 < *c* < 1, we predict the label to be *y* ^(*k*)^ = 1, otherwise *y* ^(*k*)^ = 0. A typical value for the threshold is *c* = 1/2, but it can be tuned to increase either the precision or the recall of the classifier. The fitting process identifies the parameters *θ*_*j*_ such that the classification error on the training set is minimal.

Models (1) and (2) possess three steady-state outcomes (aseptic death, septic death, and health), therefore we must go beyond the binary classification scheme described above. To classify ODE models with three steady-state outcomes, we implement the one-versus-all classification scheme. To do this, we consider three outcome labels, $y_{h}^{\left(i\right)}$, $y_{ad}^{\left(i\right)}$, and $y_{sd}^{\left(i\right)}$, for a given set of variables *x* ^(*i*)^. $y_{h}^{\left(i\right)}=0$ if the patient outcome is not health (*i.e.* aseptic or septic death) and $y_{h}^{\left(i\right)}=1$ if the patient outcome is health. Similar definitions apply for $y_{ad}^{\left(i\right)}$ and $y_{sd}^{\left(i\right)}$ (See Table 4). We use these outcome labels and Eq 20 to determine three probability functions, *P*_*h*_(*x*), *P*_*ad*_(*x*), and *P*_*sd*_(*x*). Given an unlabeled set of variables $x^{\left(k\right)}=(x_{1}^{\left(k\right)},x_{2}^{\left(k\right)},\ldots ,x_{N}^{\left(k\right)})$, we calculate *P*_*h*_(*x* ^(*k*)^), *P*_*ad*_(*x* ^(*k*)^), and *P*_*sd*_(*x* ^(*k*)^) and select the outcome with highest probability to be the predicted outcome.

**Table 4:** One-versus-all classification of outcomes. The three one-versus-all classifications for ODE models with three steady-state outcomes.

Class 1 (*y* = 0)	Class 2 (*y* = 1)
septic death + health	aseptic death
health + aseptic death	septic death
aseptic death + septic death	health

## Results

For a deterministic system of ODEs, the basin of attraction for a given fixed point is defined as the collection of initial conditions that evolve to that particular fixed point. For a given set of parameters, each of the ODE models (1)-(6) possesses well-defined (non-overlapping) basins of attraction for each fixed point.

However, different outcomes can be achieved even for a single initial condition if the parameters of the ODE model are varied. (See Fig 5). For example, the ratio of the parameters *s*_*c*_ and *μ*_*c*_ determines the background level of cortisol in model (1). Background cortisol levels are known to vary from patient to patient and can vary from one organ system to another in a given patient. To mimic these variations, we select sets of parameters randomly with mean values in Table 1 and standard deviations relative to their mean values (*i.e.* coefficient of variation) given by *v*. (See Materials and Methods). With patient variation, an initial condition can possess multiple outcomes, and thus the basins of attraction for the fixed points overlap as shown in Fig 1.

We seek to predict the patient steady-state outcomes in models (1)-(6) in the presence of patient variability *v*. For the prediction method, we employ logistic regression with one-versus-all classification [27]. (See Materials and Methods). We compare the prediction accuracy *A* at patient variability *v* to the average best guess of the steady-state outcome. For the best guess method, we determine the steady-state outcome for each of 10^2^ sets of parameters for a given initial condition. We define the best guess as the steady-state outcome with the highest number of occurrences and record the frequency *f*_*i*_ of the best guess for initial condition *i*. We then average the frequency *f*_*i*_ over 10^3^ initial conditions for each *v* to obtain an estimate for the prediction accuracy in systems with basin overlap.

For the prediction method, we solve a given system of ODEs for *N*_*i*_ = 10^4^ random initial conditions, each with randomly selected parameter sets with coefficient of variation *v*. We choose *N*_*t*_ = 800 of the *N*_*i*_ trajectories randomly to train the classifier and predict the outcome of the remaining 9200 trajectories. The classifier maps the state of the system at a given time *t*_*c*_ to a particular steady-state outcome. The prediction accuracy is then averaged over 10 training and prediction runs, each with *N*_*t*_ = 800 randomly selected training trajectories.

**Fig 6:**
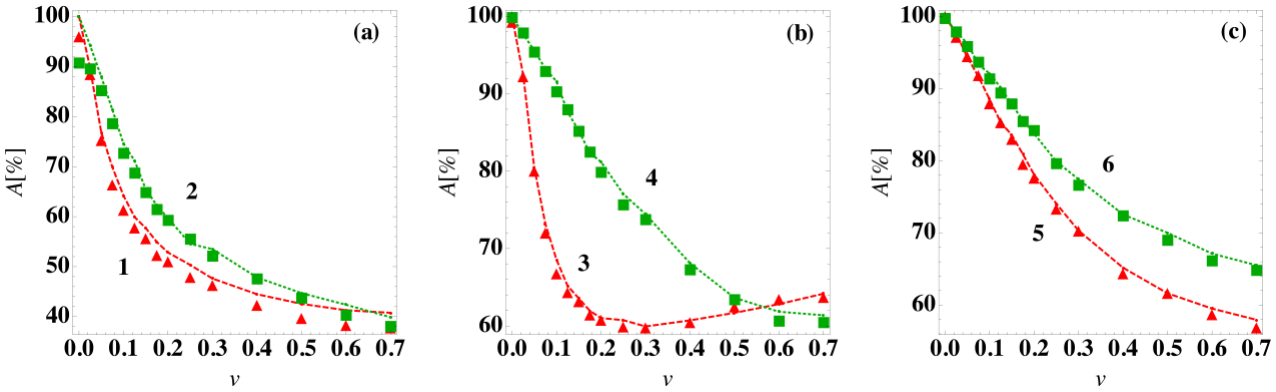
Prediction accuracy of the steady-state outcome as a function of patient variation. The prediction accuracy *A* using a logistic regression classifier at time *t*_*c*_ = 0 (symbols) and the average best guess over 10^3^ initial conditions (dashed curves) versus patient variation *v* for (a) models (1) and (2), (b) models (3) and (4), and (c) models (5) and (6).

In Fig 6, we compare the accuracy *A* of the logistic regression prediction method (with classification at time *t*_*c*_ = 0) to the average best-guess frequency as a function of the patient variability *v* for models (1)-(5). For all model ODEs, the prediction accuracy for the logistic regression prediction method is near 100% at *v* = 0, decreases for increasing patient variability, and reaches a plateau near 1/*n*_*f*_ in the large *v* limit, where *n*_*f*_ is the number of stable fixed points in the model (except for model (3)). For model (3) with two steady-state outcomes, the prediction accuracy is non-monotonic and increases for *v* > 0.3 because this ODE model begins to sample parameter regimes where one steady-state outcome is much more probable than the other. In addition, for all models the average best-guess frequency provides an upper bound for the accuracy of the prediction algorithm. Hence, the overlap of the basins of attraction imposes a limit on the prediction accuracy. To test the generality of these results, we also studied a generalized one-dimensional ODE (model (6)) with varied fixed point structure compared to that for model (5). (See Fig 3). As shown in Fig 6(c), the results for model (6) are qualitatively similar to those for models (1)-(5).

**Fig 7:**
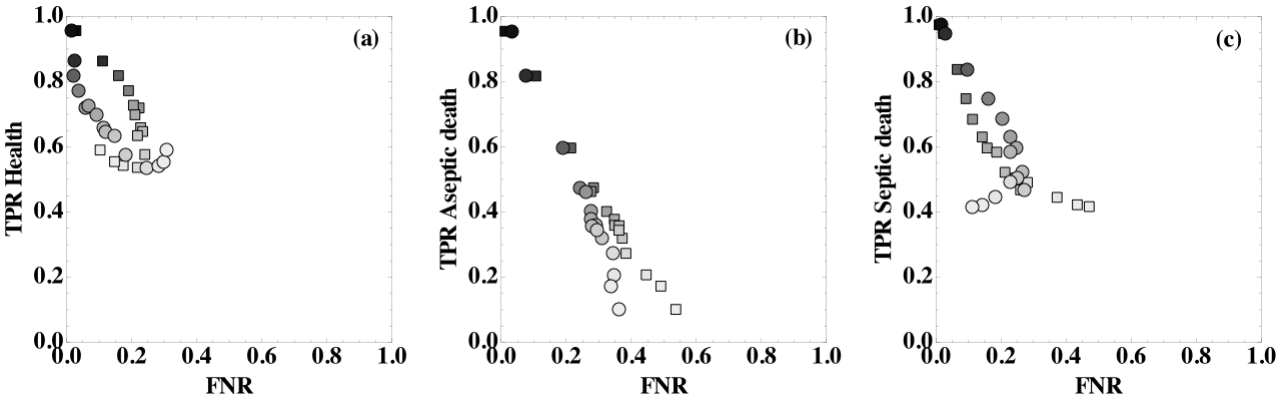
True positive rates versus false negative rates as a function of the prediction accuracy for model (1). True positive rates (TPR) for each outcome, (a) health, (b) aseptic death, and (c) septic death, versus the false negative rates (FNR) for model (1). For the FNR in (a), squares (circles) indicate that the outcome was health, but the prediction was aseptic death (septic death). For the FNR in (b), squares (circles) indicate that the outcome was aseptic death, but the prediction was health (septic death). For the FNR in (c), squares (circles) indicate that the outcome was septic death, but the prediction was health (aseptic death). The intensity of the shading of each symbol represents the prediction accuracy and scales linearly from *A* = 1.0 (black) to 0.33 (white), which is obtained from random guessing.

To obtain more detailed information about the performance of the classifier, we also studied the true positive rates (TPR) and false negative rates (FNR) for each steady-state outcome (health, aseptic death, and septic death) for model (1) (Fig 7). We find that high true positive rates are strongly correlated with high prediction accuracy *A*. As the accuracy decreases, the TPR decreases and FNR increases. Fig 7(b) also shows that one reason for low prediction accuracy at high patient variability is the difficulty of the classifier to identify aseptic death states as evidenced by the low TPR values.

**Fig 8:**
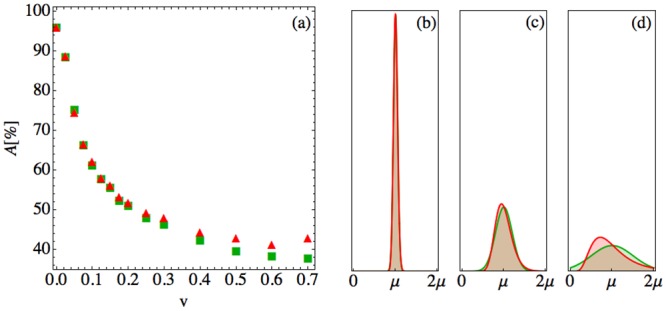
Comparison of the prediction accuracy for patient parameter variability sampled from normal and log-normal distributions. (a) Prediction accuracy *A* versus parameter coefficient of variation *v* for model (1) and classification time *t*_*c*_ = 0 with parameters sampled from a truncated Gaussian (red triangles) and log-normal (green squares) distributions. Sample Gaussian (dashed red) and log-normal (solid green) parameter probability distributions *P*(*q*) with mean *μ* and coefficient of variation (b) *v* = 0.05, (c) 0.2, and (d) 0.5. For small *v*, the Gaussian and log-normal distributions overlap. For *v* ≥ 0.5, the two distributions begin to differ near *q* = 0 and *q* ≫ *μ*.

We further investigated the influence of a different parameter sampling method on the prediction accuracy. Fig 8 compares the sampling of parameters of model (1) in Fig 6 to the sampling with a log-normal distribution instead of with a truncated normal distribution. Fig 8(a) shows the prediction accuracy versus patient variability data of model (1) in Fig 6 for sampling with truncated normal and log-normal distributions. In all cases the means and the standard deviations of the two distributions are the same. For small *v*, the distributions are very similar and hence the prediction accuracies are very similar. For increasing *v* the distributions differ more (panels (b)—(d)). Therefore the sampling of states differs, and with it there is a larger discrepancy in the prediction accuracy.

**Fig 9:**
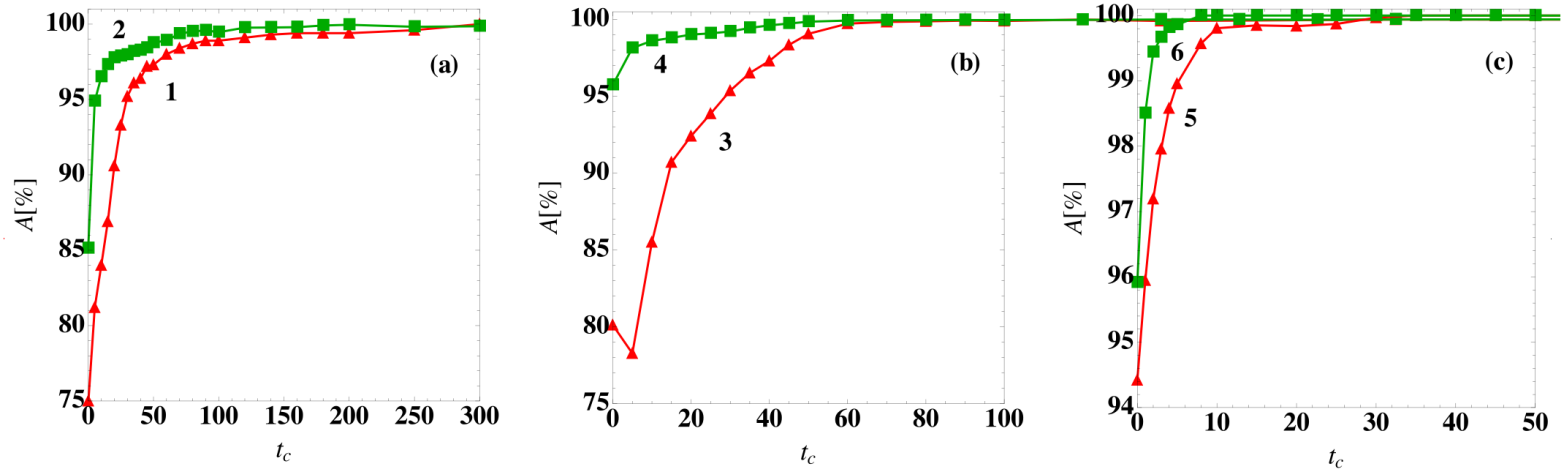
Prediction accuracy of the steady-state outcome as a function of the classification time. The prediction accuracy *A* using a logistic regression classifier at time *t*_*c*_ (symbols) for (a) models (1) and (2), (b) models (3) and (4), and (c) models (5) and (6) for patient variation *v* = 0.05.

In Fig 6, we showed results for the logistic regression prediction method with classification at *t*_*c*_ = 0. In Fig 9, we show the prediction accuracy for models (1)-(6) with patient variability *v* = 0.05 as a function of the classification time *t*_*c*_. For all models, the prediction accuracy grows with increasing *t*_*c*_, reaching nearly 100% beyond a characteristic time t* that depends on the model. The prediction accuracy improves at later classification times because the system trajectories have moved closer to the fixed points and hence the basins of attraction are more easily separated as shown in Fig 1 for model (1).

**Fig 10:**
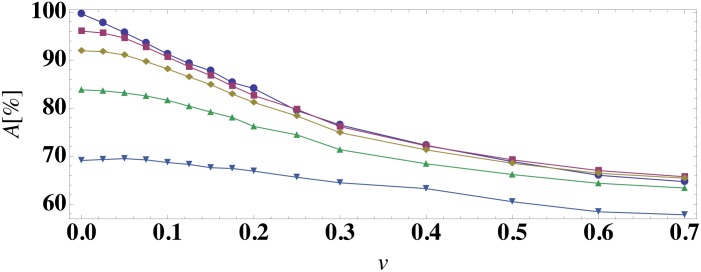
Prediction accuracy as a function of patient variation for different noise strengths. The prediction accuracy *A* using a logistic regression classifier at time *t*_*c*_ = 0 for model (6) in the presence of measurement noise with strength *s* = 0 (circles), 0.05 (squares), 0.10 (diamonds), 0.20 (triangles), and 0.50 (triangles).

We also investigated the variation of the prediction accuracy in the presence of measurement noise. We took the trajectories generated for Fig 6 and added Gaussian random noise to the model variables with variance *s* at each time point. We then performed training and testing on the noisy data with classification at time *t*_*c*_ = 0. In Fig 10, we show for model (6) that the prediction accuracy decreases with increasing *s*. We find similar results for models (1)-(5). These results emphasize that even if the measurement noise could be reduced to zero, the patient variation imposes an intrinsic limitation to outcome prediction.

## Discussion

In clinical settings it is of great importance to determine patient outcomes as quickly as possible with maximum accuracy. In this manuscript, we studied the effects of patient variability on the ability to predict steady-state outcomes in systems of ODEs that model the immune response to infection. For deterministic systems of ODEs with a given fixed set of parameters, each initial condition can be mapped to a given steady-state outcome (or fixed point) and the collection of initial conditions that map to a given steady-state outcome is defined as the basin of attraction of that outcome. Each virtual patient can be defined by a given set of parameters in the model ODE and patient variability can be introduced by varying the model parameters.

We showed that the introduction of patient variation leads to overlaps of the basins of attraction for the steady-state outcomes. In particular, a given initial condition can map to multiple steady-state outcomes for different virtual patients (*i.e.* *v* > 0), which is similar to the case of patients showing different responses to infection in clinical settings. We find that the prediction accuracy of the outcomes decreases strongly with increasing patient variability. Our results emphasize that even when the complete state of the system is known (*i.e.* all patient variables are measured precisely as a function of time), we have limited knowledge of the patient outcome when there is patient-to-patient variability that gives rise to basin overlap.

Our results also show that for all of the model ODEs studied the prediction accuracy increases as the time *t*_*c*_ used for classification increases. As *t*_*c*_ increases, the systems move closer to their steady-state outcomes and the basins of attraction separate, which increases the prediction accuracy. Again, this result is consistent with clinical experience. If a clinician waits to see if the condition of the patient improves or worsens, the prognosis will become more accurate. In our work, we explicitly show that patient-to-patient fluctuations cause a competition between early and accurate outcome prediction.

In this work, we focused on discrete steady-state outcomes (*i.e.* health or death of the patient) of the immune response to infection. However, in many biomedical scenarios, the outcomes involve continuous variables rather than discrete states. In future work, we will apply similar techniques to understand the effects of patient variability on the predictions of continuous model variables, for example, the immune response and vaccination efficacy for influenza [28].

Another important future direction is to combine outcome prediction with a treatment regimen. Treatments will depend on the predicted outcome, *e.g.* treatment for septic death would involve an antibiotic, while treatment for aseptic death would incorporate an immune suppressor. The predicted outcome can be updated based on the response of the patient to the treatment, and a new treatment can be identified based on the new prediction.

## References

[1] Koup RA, Safritt JT, Cao Y, Andrews CA, McLeod G, Borkowsky W, et al Temporal association of cellular immune responses with the initial control of viremia in primary Human Immunodeficiency Virus Type 1 Syndrome. J Virol. 1994; 68(7): 4650–4655. 8207839 10.1128/jvi.68.7.4650-4655.1994 PMC236393

[2] Guidotti LG, Chisari FV. Noncytolytic control of viral infections by the innate and adaptive immune response. Annu Rev Immunol. 2001;19(1): 65–91. 10.1146/annurev.immunol.19.1.65 11244031

[3] Janeway CA Jr, Medzhitov R. Innate immune recognition. Annu Rev Immunol. 2002; 20(1): 197–216. 10.1146/annurev.immunol.20.083001.084359 11861602

[4] Klipp E. Systems Biology in Practice: Concepts, Implementation And Application 1st ed Weinheim: Wiley-VCH; 2005.

[5] Masopust D, Vezys V, Marzo AL, Lefrancois L. Preferential localization of effector memory cells in nonlymphoid tissue. Science 2001; 291(5512): 2413–2417. 10.1126/science.1058867 11264538

[6] Pope C, Kim SK, Marzo A, Williams K, Jiang J, Shen H, et al Organ-specific regulation of the CD8 T cell response to Listeria monocytogenes infection. J Immunol. 2001;166(5): 3402–3409. 10.4049/jimmunol.166.5.3402 11207297

[7] Young D, Stark J, Kirschner D. Systems biology of persistent infection: Tuberculosis as a case study. Nat Rev Microbiol. 2008;6(7): 520–528. 10.1038/nrmicro1919 18536727

[8] Castillo-Chavez C, Feng Z. To treat or not to treat: the case of tuberculosis. J Math Biol. 1997;35: 629–656. 10.1007/s002850050069 9225454

[9] Perelson AS, Nelson PW. Mathematical analysis of HIV-1 dynamics in vivo. SIAM Rev. 1999;41(1), 3–44. 10.1137/S0036144598335107

[10] Callaway DS, Perelson AS. HIV-1 infection and low steady state viral loads. B Math Biol. 2002;64(1): 29–64. 10.1006/bulm.2001.0266 11868336

[11] Nelson PW, Perelson AS. Mathematical analysis of delay differential equation models of HIV-1 infection. Math Biosci. 2002;179(1): 73–94. 10.1016/S0025-5564(02)00099-8 12047922

[12] Perelson AS. Modeling viral and immune system dynamics. Nat Rev Immunol. 2002; 2(1): 28–36. 10.1038/nri700 11905835

[13] Perelson AS, Neumann AU, Markowitz M, Leonard JM, Ho DD. HIV-1 dynamics in vivo: Virion clearance rate, infected cell life-span, and viral generation time. Science. 1996:271: 1582–1586. 10.1126/science.271.5255.1582 8599114

[14] Bocharov GA, Romanyukha AA. Mathematical model of antiviral immune response III. Influenza A virus infection. J Theor Biol. 1994;167(4): 323–360. 10.1006/jtbi.1994.1074 7516024

[15] Belz GT, Wodarz D, Diaz G, Nowak MA, Doherty PC. Compromised influenza virus-specific CD8+-T-cell memory in CD4+-T-cell-deficient mice. J Virol.2002;76(23): 12388–12393. 10.1128/JVI.76.23.12388-12393.2002 12414983 PMC136883

[16] Dowdy DW, Chaisson RE, Moulton LH, Dorman SE. The potential impact of enhanced diagnostic techniques for tuberculosis driven by HIV: A mathematical model. Aids 2006; 20(5): 751–762. 10.1097/01.aids.0000216376.07185.cc 16514306

[17] Wodarz D, Nowak MA. Mathematical models of HIV pathogenesis and treatment. BioEssays. 2002;24(12): 1178–1187. 10.1002/bies.10196 12447982

[18] Serdobova I, Kieny MP. Assembling a global vaccine development pipeline for infectious diseases in the developing world. Am J Public Health. 2006 9; 96(9): 1554–1559. 10.2105/AJPH.2005.074583 16873743 PMC1551949

[19] Wetterstrand KA. DNA Sequencing Costs: Data from the NHGRI Genome Sequencing Program (GSP) Available at: www.genome.gov/sequencingcosts. Accessed Jan. 2015.

[20] Day J, Rubin J, Vodovotz Y, Chow CC, Reynolds A, Clermont G. A reduced mathematical model of the acute inflammatory response II. Capturing scenarios of repeated endotoxin administration. J Theor Biol. 2006;242(1): 237–256. 10.1016/j.jtbi.2006.02.015 16616206

[21] Vodovotz Y., Csete M., Bartels J., Chang S., & An G. Translational systems biology of inflammation. PLoS Comput Biol. 2008;4(4): e1000014 10.1371/journal.pcbi.1000014 18437239 PMC2329781

[22] Vodovotz Y, Constantine G, Rubin J, Csete M, Voit EO, An G. Mechanistic simulations of inflammation: Current state and future prospects. Math Biosci. 2009;217(1): 1–10. 10.1016/j.mbs.2008.07.013 18835282 PMC2667966

[23] Webb GF, D’Agata EMC, Magal P, Ruan S. A model of antibiotic-resistant bacterial epidemics in hospitals. P Natl Acad Sci USA. 2005;102: 13343–13348. 10.1073/pnas.0504053102 PMC1201589 16141326

[24] Wang K, Bhandari V, Chepustanova S, Huber G, Stephen O, Corey SO, et al Which biomarkers reveal neonatal sepsis?. PLoS One 2013;8(12): e82700 10.1371/journal.pone.0082700 24367543 PMC3867385

[25] O’Hara S, Wang K, Slayden RA, Schenkel AR, Huber G, O’Hern CS, et al Iterative feature removal yields highly discriminative pathways. BMC Genomics. 2013;14(1): 832 10.1186/1471-2164-14-832 24274115 PMC3879090

[26] Reynolds A, Rubin J, Clermont G, Day J, Vodovotz Y, Bard Ermentrout G. A reduced mathematical model of the acute inflammatory response: I. Derivation of model and analysis of anti-inflammation. J Theor Biol. 2006;242(1): 220–236. 10.1016/j.jtbi.2006.02.016 16584750

[27] Hastie T, Tibshirani R, Friedman J. The elements of statistical learning. New York: Springer; 2009.

[28] Hancioglu B, Swigon D, Clermont G. A dynamical model of human immune response to Influenza A virus infection. J Theor Biol. 2006;246: 70–86. 10.1016/j.jtbi.2006.12.015 17266989

